# Surgical Notes

**Published:** 1851-09

**Authors:** Clinton Colegrove

**Affiliations:** Sardinia, N. Y.


### ART. III. — *Surgical Notes.* By Clinton Colegrove, M. D.

Surgical operations may be justly arranged in two classes. 1. Of *necessity*. 2. Of *expediency*. Operations of the first kind are performed to save life, or to rescue the body from vital derangements. Those of the second, to assist nature in achieving curative results, to abolish incumbrances, and to obviate both inconvenience and deformity. The exact demarcation between the two is not readily detailed. They are often insensibly intercurrent. The decision of the surgeon upon the question of an operation, is often reached with hesitation, and his own procedure regulated by circumstances not strictly expressed in the face of a malady, especially the patient's own inclination.

Amputation, excision of malignant growths, cutting for strangulated hernia, extraction of foreign bodies, of large dimensions, or in perilous situations, ligature of bleeding vessels, trephining, bronchotomy, and a few other operations, never admit a discussion of their propriety, when the circumstances arise which ordinarily and obviously demand their performance. Nor need this be suspected a tautology, for even the most deliberate, and clear-sighted surgeons are sometimes puzzled to declare, if an operation be either necessary or expedient. (Though of these major operations I may parenthesize, that if expedient, they are necessary.) Thus an old compound fracture, badly united, with a large tract of necrosed bone and extensive ulcerations of the surrounding soft parts, is one of the most embarrassing condition of things in which a conservative surgeon can be placed, especially if to him is assigned the duty of forecasting the future of disease, without an operation, or deciding what shall be done. The progress of restoration may be tolerably rapid, yet at a critical point of time the pulse quickens, the systemic energy trembles and flags, and all determination is set at a poise. The veteran surgeon well knows what a perplexity of dumb debate he here encounters. "If I remove the limb," he argues, "and when I have dissected it, find evidence of advancing integrity, which a little procrastination would perfect, with the probable re-invigoration of life which begins at a crisis, I shall have compunctions. If I wait till hectic is too decided, it may be too late." So again, if a man have a carcinomatous growth proceeding slowly, and is himself at about the natural period of life, what shall you tell him? Ordinarily submit the question of an operation, to himself, without doubt.

Operations of *expediency*, are well exemplified in plastic surgery, in talipes, in labium leporinum, in strabismus, and you may say even in cataract. A man may stub along on club-foot, with an orbit whose visual center is occluded, with a sarcomatous tumor, with a horn on his forehead, or a peduncle of bone on his foot, and flourish as long as if he were disencumbered by the surgeon's knife. Admit degrees of expediency of course.

Improvements in surgery for the last forty years, have been mainly of the second growth. Even flap amputations date there origin as far back as that one of the most famous of all years, 1769. In the years 1813 and 1838, respectively, did the devices for supplying new features to fill up the gapes dug by disease in the human face, and for curing strabismus, begin to have celebrity. These improvements harmonize with the other discoveries of the age. Necessity, the mother of inventions, has always had a respectable fecundity. But in modern times her fruitfulness is rivalled by the magnificent productions of another dame — Enterprise. But the climax of her liberality to the therapeutic world, lies in that signal boon which permits the candidate for surgic intercession, to quaff the enchanting vapor which extinguishes pain. What an inestimable beneficence of Providence! What a mystery of pity for mortal pangs, in the heart of Divinity, disclosed now after the lapse of fifty-eight hundred and fifty years from Adam! How notably coincident with that element, which, at command, conducts the epistleship of a restless people, and with sublime velocity carries a thousand messages, as many different ways in a moment. The one obeys intelligence, the other suspends it. The one enhances and exalts the ecstacy of social existence, the other imparts the charm of unconsciousness, in an hour of private anguish.

So far have I premised, by suggestions imperfectly apologetic of these brief annotations which I append.

I was called, May 13, to perform amputation of the leg of an Irishman, at the alms-house of Cattaraugus county. Suspecting some difficulty in determining what ought to be done, I invited Dr. Shepherd, of Yorkshire, to accompany me, of whose opinion I wished to avail myself, together with that of the attending physician, Dr. Copps, of Machias. (My father was detained at home from illness.) We found the patient, æt. about 37, with a diseased ankle, much swollen, immovable without great pain, and with a number of sinuous ulcerations opening at different points, but none of them suffering a probe to reach the cavity of the joint. There was evidently a chronic thickening and solidity of all the structures in the region. Patrick had been a canal-digger with his feet much of the time submerged, to which he directly traced the disease which had origin more than a year previous. He had suffered much and almost incessant pain. There was no appearance of progress toward recovery, but the patient was slowly sinking. Pulse 110. Noticed cough, with purulent looking expectoration. Tried to see if the sputa would sink or float in an aqueous medium. It would do neither, but hung suspended just below the surface. I doubt the efficiency of this test for determining the quality of expectoration. The fact is, *ordinarily*, I take it, there is an admixture of *mucus and pus*, in cases of tubercular softening, and the sputa will not sink. Percussion did not reveal much relative dullness. On auscultation detected an exaggerated murmur on one side — little, if any, on the other. Now what shall be done? Patrick is sinking. He importunes us to remove his leg. Can he recover with it on? The answer is ready — no. Will he recover with an operation? Exceedingly doubtful. The pulmonary wasting will carry him off. Remove the diseased mass, and you have done well — but let us see. According to late and high authority,* "it seems probable that disease of the lungs, or mesentery, is sometimes suspended or averted, by the continuance of a disease in the extremity." But again, "a *severe* outward disease might induce the very same disease in the lungs or mesentery, which a more moderate degree might avert. Recovery without amputation, is far more probable, when one of the larger articulations is affected, than when the complicated joints of the tarsus or carpus are involved."

Decided to operate. Undertook to delineate my course with nit. argenti, but the stain being too tardily developed, was obliged to use a more effaceable material. Elevated the leg sufficiently long to be evacuated of nearly

* Druitt's Surgery, p. 272.

all the venous blood, and the hæmorrhage did not trouble my landmarks. Chose the *second place of election* of Pancoast, or middle of the leg, and followed my father's usual custom of making *lateral flaps.* With this method he has always been satisfied, notwithstanding the almost universal preference given to a single and posterior flap. The advantage of a convenient point for the egress of pus or blood, at the inferior and depending part of the stump, is not valueless. Neither does he operate by transfixion, since on trial, he was incommoded by muscular spasm, which impaired the symmetry of the flap. After sawing the bones, (I removed the anterior edge of the tibial extremity,) the flaps coincided well.

The application of ligatures was troublesome, since, the elasticity of the arteries being impaired, the usual exit of blood, *per saltum*, did not occur. From works on surgery, we are taught, I believe, to depend on the eye, for the detection of the arterial orifice. May I make bold to suggest the propriety, now manifest to me, of trusting *to the touch*, for finding the retreated vessel. Its somewhat hard and horny feel, would, I imagine, greatly assist in its discovery and seizure.

Retained the flaps in juxtaposition, by adhesive straps. I soon regretted that I did not use sutures. I think they would better secure quick and certain union. Saw the patient no more. At the expiration of five weeks he succumbed, the wound not having entirely united. Did not administer chloroform, as it was unwarrantable. Comments are unnecessary, the case illustrating observations in the former part of this paper. I will merely add that from symptoms and subsequent dissection, the disease was pronounced to be cartilaginous ulceration of the joint.

If not irrelevant, I will give the notes of two cases of paraphymosis. The first occurred to a lad of five years, who, after retracting the prepuce, could not return it. After twelve hours, the father tried reduction of the glans without success. Believing it necessary after a short trial of taxis to divide the constricting portion of the prepuce, I introduced a sharp-pointed, curved bistoury, and after several sections at different points, succeeded. In the second case, a young man recently married, had the misfortune, (the prepuce slipping behind the glans *in coitu*,) to be unable to return it. A natural delicacy restrained him from a disclosure of the fact to a physician, until the lapse of five days, when the great and alarming tumefaction, which supervened together with pain, and ulceration at the point of the stricture, impelled him to apply for relief. Dr. S. attempted reduction, after the faithful use of cold water, which considerably reduced the swelling, but was induced to desist. The next day, I saw the patient, and proceeded to attempt his relief.

This was with difficulty accomplished, and only after repeated and cautious incisions, and protracted extension and taxis, — and then imperfectly, — the surrounding infiltration rendering the perfect restoration of parts to their natural position, impracticable. This, in neglected cases, we are taught to expect.

Truly yours,

C. COLEGROVE.

SARDINIA, N. Y., July 18, 1851.

